# Suggested Guidelines for the Treatment of Mycosis Fungoides in Countries with Limited Resources

**DOI:** 10.1155/2023/1360740

**Published:** 2023-01-31

**Authors:** Mona Abdel-Halim Ibrahim, Nada Eltayeb, Mirna Michel Ibrahim, Ahmed Nassar, Maged Daruish, Maha El-Zimaity, Mahmoud El-Lithy, Ahmed Mostafa, Amal El-Afifi, Haitham Abdelbary, Mahira Hamdy El-Sayed

**Affiliations:** ^1^Dermatology Department, Ain-Shams University, Cairo, Egypt; ^2^Department of Dermatopathology, St. John's Institute of Dermatology Guy's and St. Thomas' Nhs Trust, London, UK; ^3^Hematology Department, Ain-Shams University, Cairo, Egypt; ^4^Oncology Department, Ain-Shams University, Cairo, Egypt

## Abstract

The treatment options for mycosis fungoides (MF) have been expanding but unfortunately many of the currently used treatment modalities are unavailable in Egypt and other African/Arab countries. In addition, there is a lack of consensus on the treatment of hypopigmented MF (HMF), which is a frequently encountered variant in our population. We aimed to develop regional treatment guidelines based on the international guidelines but modified to encompass the restricted treatment availability and our institutional experience. Special attention was also given to studies conducted on patients with skin phototype (III-IV). Treatment algorithm was formulated at Ain-Shams cutaneous lymphoma clinic through the collaboration of dermatologists, haematologists, and oncologists. Level of evidence is specified for each treatment option. For HMF, phototherapy is recommended as a first line treatment, while low-dose methotrexate is considered a second line. For early classical MF, we recommend Psoralen-ultraviolet A (PUVA), which is a well-tolerated treatment option in dark phenotype. Addition of either retinoic acid receptor (RAR) agonist and/or methotrexate is recommended as a second line. Total skin electron beam (TSEB) is considered a third-line option. For advanced stage, PUVA plus RAR agonist and/or methotrexate is recommended as first line, TSEB or monochemotherapy is considered a second line option. Polychemotherapy is regarded as a final option. All patients with complete response (CR) enter a maintenance and follow-up schedule. We suggest a practical algorithm for the treatment of MF for patients with dark phenotype living in countries with limited resources.

## 1. Introduction

Mycosis fungoides (MF) is the most common type of cutaneous lymphoma. It has an indolent chronic course refractory to various therapies. Typically, MF presents in its early stages with patches and plaques that may progress to tumours, erythroderma, or visceral involvement, with poor prognosis (Supplementary Tables 1–3 show the ISCL/EORTC revision of the classification of MF/SS (2007), the histopathologic staging of lymph nodes in MF/SS, and the ISCL/EORTC revision to the staging of MF/SS, respectively) [1–4]. Of the many MF subtypes, hypopigmented MF (HMF) is of interest due to its high prevalence among the darker skin type of our population. Apparently, the contrast of the hypopigmented patches makes the diagnosis easier; yet, the true prevalence among different skin types needs further studies. It is frequently seen in younger age group and is often associated with CD8^+^ profile with an indolent course and a high recurrence rate [5]. On the other hand, some variants may have an aggressive behavior as follicular MF (FMF) and MF with large cell transformation (LCT). Therefore, treatment options should be tailored according to the clinical and the histopathological features of the disease [6, 7].

Management of MF is based on its stage. For early stages, therapies include topical corticosteroids, phototherapy, topical bexarotene, radiotherapy, and nitrogen mustard preparations. While for advanced stages, options include bexarotene, histone deacetylase inhibitors, interferon, antibody therapies, systemic chemotherapy, and allogeneic hematopoietic cell transplantation (HCT) [8].

Many of the current mainstay treatment options for MF, such as bexarotene, extracorporeal photophoresis, interferon, histone deacetylase inhibitors, nitrogen mustard, and biologics are either unavailable or not covered by medical insurance in Egypt and other countries with limited resources [9]. In addition, there is no standardized stepwise approach among different specialties in the management of cutaneous lymphoma; certain specialties might use aggressive treatment lines in early stages of the disease which increase the risk of relapse and the mortality rate. Thus, management of MF with adherence to international guidelines represents a major challenge. We therefore present an alternative treatment algorithm for adult patients with MF and patients with HMF of all ages with special consideration to darker skin phototypes, excluding patients with other types of systemic lymphoma if associated. The presented guidelines are based on our institutional experience and the treatment availability in our country. This is formulated to provide a practical guide for dermatologists, haematologists, and oncologists to unify the quality of care in clinics and institutions all over Egypt and to share our experience with other countries with limited resources.

## 2. Methods

Treatment approach was based on the literature review for the international guidelines for the treatment of MF as European Organisation for Research and Treatment of Cancer (EORTC), 2017 [10]; European Society for Medical Oncology (ESMO), 2019 [11]; National Comprehensive Cancer Network (NCCN), Version 2 2019 [12] and 2020 [13]; and British Association of Dermatologists and U.K. Cutaneous Lymphoma Group guidelines (BAD-UKCLG), 2018 [14]. The literature review was done until July 2020 on PubMed, Embase databases, the Cochrane Library for meta-analysis, systemic reviews, randomized and nonrandomized clinical studies, cohort, case-control studies, case series, and case reports of MF and Sezary syndrome (SS) treatment options using specific search terms shown in (Supplementary Table 4). Special attention was also given to studies conducted on patients with skin phototype (III-IV). Only English articles with available/accessible treatment modalities were included. This guideline has been developed with reference to the Appraisal of Guidelines Research and Evaluation (AGREE II) instrument (http://www.agreetrust.org) [15].

The guideline development group included dermatologists, haematologists, and oncologists at Ain Shams cutaneous lymphoma clinic. Different authors were allocated to the appraisal of the literature review of specific treatment options. The guideline was discussed among patients attending the cutaneous lymphoma clinic, using previously formulated self-assessment questionnaires, phone calls, and face to face interviews, to gather their opinions regarding the efficacy, cost, feasibility of each treatment, and its effect on their quality of life. The guideline has been peer reviewed by dermatology consultants with special expertise in the field of cutaneous lymphoma to assess its usefulness and applicability. Recommendation drafts were sent through e-mails and were circulated to the authors for final amendments to be made. Informal consensus was reached through a series of discussions and several e-mail deliberations.

Recommended treatment options are presented as an algorithm according to the disease stage. Patients with specific variants are discussed separately owing to the variation in their prognosis from classic MF. Dose and treatment duration are provided; and maintenance/follow-up protocol is designed. Level of evidence is specified for each treatment option using Oxford Centre for Evidence-Based Medicine 2011 (OCEBM) (Supplementary Table 5 shows the details of OCEBM) [16].

Data regarding the response rate, treatment availability, cost, side effects, emerging new drugs, patients' preferences, and quality of life will be collected and registered in the clinic database.

This guideline will be reviewed and updated every 5 years if deemed necessary depending on the results of any future studies and collected data.

## 3. Results

### 3.1. Treatment Options for Classic MF

#### 3.1.1. Expectant Therapy

Expectant therapy has been recommended by the EORTC for patients with stage IA MF especially those with T1a because they usually show very low risk of progression (10% after 10 years) with normal life expectancy [10, 17–19]. However, expectant therapy is not convenient in our community due to lack of some patients' commitment to regular follow-up. In addition, Pavlotsky et al. [20] reported that progression and even rare fatalities may occur in early-stage disease.

#### 3.1.2. Topical Treatment

Available topical treatment options are listed in Table 1.

*(1) Topical Steroids*. Topical corticosteroids are widely accepted for treatment of MF, either as monotherapy in stage IA or adjuvant therapy in later stages. Clobetasol propionate cream 0.05% applied twice daily show remission; however, responses are not durable or complete [11, 21]. Side effects from prolonged use are minimal [22].

*(2) Topical Retinoids*. Bexarotene is a retinoid *X* receptor that is approved by the FDA for the treatment of refractory cutaneous lesions in stage I [23]. Unfortunately, it is not available in Egypt and instead, we use tazarotene 0.1% gel for refractory patches [24]. It has been studied for refractory lesions and shows promising results. It can also be used under occlusion combined with topical steroid ointment [25]. Moreover, in a Canadian study, tazarotene 0.1% cream was used as monotherapy for early patch disease [26]. It is important to mention that like bexarotene, tazarotene is contraindicated during pregnancy.

*(3) Topical Chemotherapy*. Topical mechlorethamine was approved by the European Medicines Agency (EMA) for the treatment of early-stage MF but it is not available in our country [27]. An interesting *in vitro* and *ex vivo* study showed that gentian violet, the widely available inexpensive agent, can act as anticutaneous lymphoma agent through inducing tumour cell apoptosis and blocking its growth [28]. In addition, there is a case report of a patient with recalcitrant, localized patch disease stage IB that showed improvement and reduction of erythema with gentian violet when applied once daily for 2 months [29]. Therefore, we included this agent in our guidelines.

*(4) Moisturizers*. Moisturizers are used as basic routine care for patients with MF. It is of pivotal importance to maintain the integrity of skin barrier in these patients to guard against infections [30]. They may reduce transepidermal water loss and decrease the scaling and itching sensation [31]. Their role is highlighted in a placebo-controlled study evaluating the purine nucleoside phosphorylase inhibitor (peldesine cream) for the treatment of cutaneous lymphoma. Results showed relatively high (24%) placebo response rate versus (28%) the peldesine group [32].

*(5) Other Topical Therapies*. Other studies investigated the use of imiquimod [33, 34], tacrolimus [35], and 5-fluorouracil [36], and show their beneficial role in the treatment of early MF; however, there is a lack of controlled studies to validate their efficacy; therefore, we did not include them in our guidelines [31].

#### 3.1.3. Phototherapy

*(1) Narrow-Band Ultraviolet B (NB-UVB)*. NB-UVB is the most readily available type of phototherapy in Egypt. It can cause complete remission of patches ranged from 54% to 90% [37, 38]. A Tunisian study showed that NB-UVB is effective for the treatment of noninfiltrative plaque irrespective of the skin phototype [39]. NB-UVB is administered as 3 sessions per weeks in most of the studies [38]. Studies did not show increased carcinogenesis with NB-UVB [38, 40]. Interestingly, patients who have not previously responded to psoralen-ultraviolet A (PUVA) may show improvement with NB-UVB [38, 41, 42].

*(2) Psoralen-Ultraviolet A (PUVA)*. PUVA is an effective treatment for early MF especially in patients with thick plaques, dark phenotypes, and those refractory to NB-UVB [38, 43, 44]. It is more effective than NB-UVB in inducing complete clearance [8, 45]. The NCCN [13] and BAD [46] recommend PUVA as a first line for predominately plaque disease. Durable remission (10 years) can be achieved in 30–50% of patients [47]. Complete response (CR) is achieved in 85% for stage IA, 65% for stage IB, and 85% for stage IIA. The time to CR is much longer in patients with plaques than patches [38]. An Egyptian study showed that patients respond less to phototherapy and usually need double the number of sessions due to their darker phenotype [48]. PUVA is often prescribed with 8-methoxypsoralen (MOP), that is available in Egypt and given 2-3 times weekly [31]. High cumulative dose of PUVA can lead to photodamage and photocarcinogenesis. Lifetime PUVA exposure should be limited to 1200 J·cm^2^ and/or 250 sessions [46]; however, these studies have been performed on patients with fair colour. No studies are available from populations with darker phototypes that are naturally more protected against photodamage.

Regarding the tumour stage, PUVA is given combined with systemic therapy as CR to monotherapy for most of the studies is poor. In erythroderma, CR is achieved in 43%; however, patients only tolerate low doses of PUVA owing to photosensitivity [38].

Bath PUVA is not commonly used and can lead to early relapse as the head is not exposed to treatment. However, it can be reserved for patients with contraindications to oral PUVA. Treatment was given at a dose of 0.2 mg/L 8-methoxypsoralen, 3 times weekly followed by UVA irradiation [43, 49, 50]. Topical PUVA can be given to unilesional MF or pagetoid reticulosis [51].

Studies on phototherapy for MF patients are shown in Table 2.

For decades, climatotherapy has been considered as an established line of treatment for psoriasis. Recently, it has been suggested for other T cell-mediated skin conditions, like mycosis fungoides and atopic dermatitis. Several locations have been suggested as destinations for climatotherapy, including the alpine mountains in Switzerland, the Dead Sea, and certain locations on the Red Sea. Luckily, the latter is available in Safaga, Red Sea, Egypt. Direct exposure to the sun on a regular basis for several days showed some promising results in inducing remission in patch-stage MF. In addition, PUVA-sol has been suggested as an alternative home-based phototherapy in some regions. However, this treatment modality needs to be studied further. It should be considered as a complementary modality rather than a treatment on its own [56, 57].

#### 3.1.4. Phototherapy Plus Systemic Therapy

PUVA can be combined with systemic treatment as interferon and retinoids to increase the efficacy in refractory/advanced cases and to decrease the cumulative dose of UVA, thus, reducing the long-term side effects. According to ESMO guidelines, it is considered as a 1^st^ line in the advanced stage and a 2^nd^ line therapy in refractory cases of the early stage [11]. However, sufficient data regarding the efficacy of combined treatment over PUVA monotherapy are lacking [38].

Drugs available in Egypt that can be combined with PUVA are retinoic acid receptor (RAR) agonists and methotrexate.

*(1) PUVA and RAR Agonists*. RAR agonists include acitretin and isotretinoin. Thomson et al. [58] treated a group of early MF patients with either etretinate or isotretinoin plus PUVA and the other group was treated with PUVA alone. The response rate did not significantly differ between both groups; however, the dose of UVA is much lower in the combined group which decrease the toxicity. In addition, patient showed a prolonged remission when they receive retinoids as a maintenance therapy. In a recent multicentre retrospective study in Greece, acitretin was shown to be more effective when patients concomitantly received PUVA or topical steroids than when patients receiving acitretin alone. Most of the patients were at early stage (92%), and 18% had FMF [59].

*(2) Phototherapy and Methotrexate*. The combination of PUVA and methotrexate is not recommended due to the risk of increase carcinogenesis [38]. However, no solid evidence exists regarding this risk especially in populations with darker phenotype. This combination has been used in psoriasis and proved its efficacy [60, 61] and antiangiogenic effect [62]. Moreover, recent mathematical modeling of cancer cell mutational dynamics hold a new promise for the use of methotrexate in combination with NB-UVB through attacking multiple signaling pathways simultaneously. This will decrease cross resistance and provide better disease control [63].

#### 3.1.5. RAR Agonists as a Systemic Monotherapy

RAR agonists exert a modest activity against MF as a monotherapy with more potent effect when combined with phototherapy [14, 59]. In a retrospective study on patients with early MF, 25 patients received acitretin and 12 patients received isotretinoin at a dose of 0.3 mg/kg and 0.2 mg/kg, respectively. The median treatment duration was 10 months for acitretin, and 9 months for isotretinoin. The overall response was 64% for acitretin and 80% for isotretinoin, and CR was 4% and 8%, respectively [64].

Cumulative data regarding the use of RAR agonists in MF showed that the overall response rates ranges from 43% to 100% and the median response duration ranges from 3 to 15 months [65]. No controlled studies are available comparing the efficacy of acitretin versus isotretinoin [10]. Nevertheless, a single case report showed the superior efficacy of isotretinoin for the treatment of FMF [66]. According to the NCCN guidelines 2020, acitretin is regarded as an alternative category A systemic treatment and can be used starting stage IIB [13].

#### 3.1.6. Other Combined Systemic Treatments

Methotrexate enhances the effect of bexarotene [67]. Methotrexate and acitretin have been used before in psoriasis in refractory cases and showed efficacy with no report of increased liver toxicity [68]. However, no enough data exists regarding the combined use of methotrexate and acitretin for MF.

#### 3.1.7. Radiotherapy

**Figure 1:**
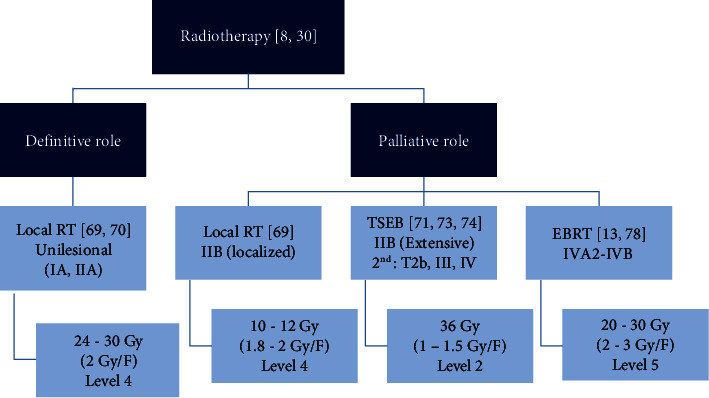
Protocol of radiotherapy treatment for MF/SS in Egypt. Localized radiotherapy is given to unilesional MF or nodular-stage MF with few nodules. TSEB is given to nodular-stage MF with extensive nodules or as a second-line therapy for resistant plaques, erythroderma, or stage IV. EBRT can be given in case of metastasis. RT: radiotherapy, TSEB: total skin electron beam, EBRT: external beam radiotherapy.

Radiotherapy is considered an important treatment option in the management of patients with MF in both early and advanced stages [69], and can be used for the eradication of unilesional disease or for the palliation of multisite disease. Total skin electron beam (TSEB) may be used for effective palliation, and those patients may often have long-term disease-free intervals [70], but we have to consider that due to the rarity of this disease, there are no randomized trials directly comparing radiotherapy to other treatment options [69]. Protocol of radiotherapy treatment for MF/SS in Egypt is shown in Figure 1.

*(1) Radiotherapy in Unilesional Mycosis Fungoides*. Radiotherapy can be curative in patients with unilesional MF and CR rates can be as high as 100%, with no recurrences at treated sites [71]. In the few available reports, doses have ranged between 6–40 Gy (usually 1.8 Gy/F) with local recurrence unusual above 24 Gy [70]. The usual dose used in Ain Shams University ranges between 24 and 30 Gy/F usually using a tissue equivalent bolus (thickness either 0.5 or 1 cm) with 2 cm margin.

*(2) Localized Radiotherapy as a Palliative Measure in MF*. Radiotherapy plays a central role in local palliation, particularly for lesions localized in sanctuary areas. Symptomatic cutaneous lesions (as cosmetic disfigurement, itching, scaling, or discharging) and lesions unresponsive to other therapeutic options may be managed with radiotherapy in any stage of MF [72].

Low doses as 4 Gy (2 Gy/F) can be used and allows overlapping fields to treat lesion at any site [69], but response rate is low being <30% and so higher palliative doses (8 Gy or more) are recommended, doses ranging between 8 and 12 Gy allow retreatment of needed lesions [70]. The usual palliative doses used in Ain Shams University ranges between 10 and 12 Gy (2 Gy/F) using a tissue equivalent bolus as in unilesional treatment but a narrower margin may be used. It is usually given to patient with localized nodules (stage IIB).

*(3) Total Skin Electron Beam (TSEB)*. Modern TSEB has an overall response rate approaching 100% and remains a fundamental treatment for MF, with no other treatment approaching such a high response rate. However, it is a very complicated treatment requiring a skilled multidisciplinary team, highly experienced in the management of cutaneous lymphoma [73]. These technical difficulties and the unavailability of radiotherapy unit that is constructed for offering TSEB in Ain Shams University represent the main obstacle in applying TSEB as a routine management of patient with MF and so we usually refer our patient to receive TSEB (whenever indicated) to other radiotherapy units (although limited number of these units in Egypt generally). It is usually given to patients with stage IIB with extensive nodules and as a second line therapy for patients with T2b, stage III and IV.

According to EORTC guidelines [74], TSEB can be used for patients with all stages of MF, and remains a very important treatment for these patients, even for those with SS. Moreover, the recent NCCN guidelines 2020 stated that TSEB can be considered as early as stage IB [13]. The response rates and duration of response are higher in earlier stage disease. The aim of treatment (curative or palliative) varies depending on the stage [69]. The goal of TSEB is to deliver a relatively uniform dose of radiation to the entire skin while limiting acute and long-term toxicities [75].

**Figure 2:**
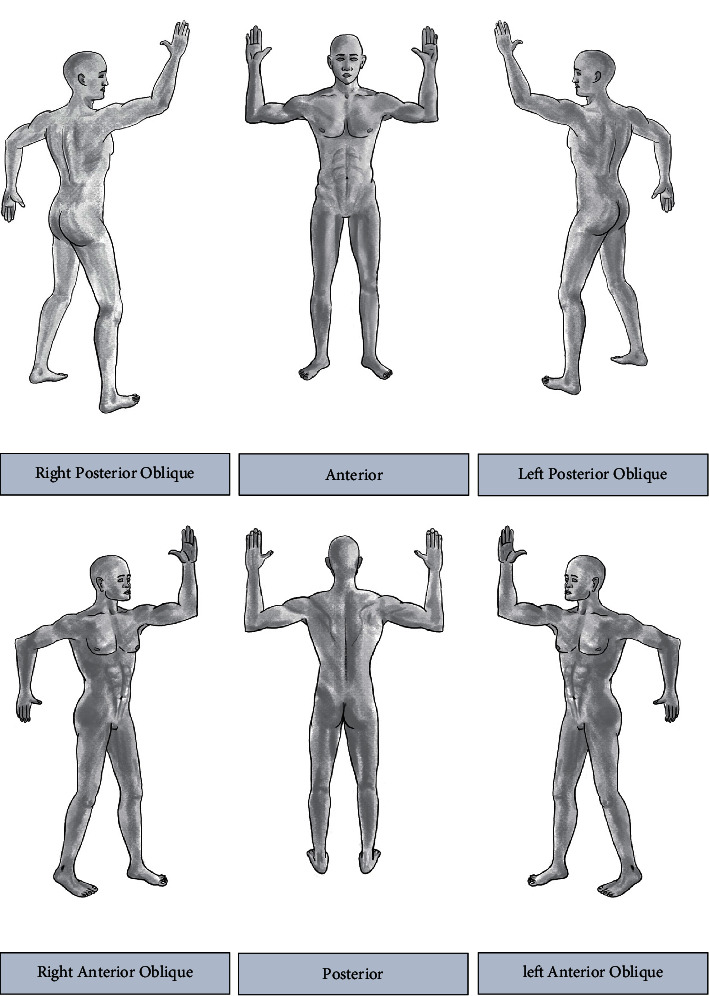
Patient positions for total skin electron beam therapy, 6-field technique. The straight anterior, right posterior oblique, and left posterior oblique fields are treated on one day. The straight posterior, right anterior oblique, and left anterior oblique fields are treated the next day [69].

The 6-field large electron field technique developed at Stanford is the most commonly used [76]. The patient is treated in six different standing positions over the course of 2 treatment days (Figure 2) [75]. This cycle is repeated twice per week [75], the traditional dose used is as high as 36 Gy [74], and still this is our recommended dose in Ain Shams University whenever referring a patient with MF for TSEB.

More recently, lower dose regimens (10–12 Gy) have been investigated and showed their efficacy with the advantage of allowing multiple retreatments and being more convenient for patients [77, 78]. However, there are no controlled comparative studies investigating the efficacy of the standard dose versus the low dose TSEB in inducing remission [10].

*(4) External Beam Radiotherapy in Metastatic Disease*. External beam radiotherapy can be given for patients with nodal or visceral involvement as the standard approach for patients with non-Hodgkin lymphoma in a dose 20–30 Gy (2-3 Gy/F) [14, 79].

#### 3.1.8. Chemotherapy

According to NCCN 2020 guidelines, methotrexate ≤50 mg per week is considered as category A systemic treatment for treatment of patients with tumour stage. Category B systemic treatment, which are more toxic include Gemcitabine and pegylated liposomal doxorubicin can be used for patients with advanced stage MF/SS. Multiagent chemotherapy, though effective, are more toxic and associated with higher risk of mortality, so are reserved only for refractory cases or for nodal or visceral metastasis. They can also be used as a bridge to allogeneic HCT [13]. Methotrexate can be used from stage IIB, gemcitabine or liposomal doxorubicin can be used from stage IV and polychemotherapy is regarded as a final treatment option (Table 3).

### 3.2. Algorithm for Treatment of Classic MF/SS

**Figure 3:**
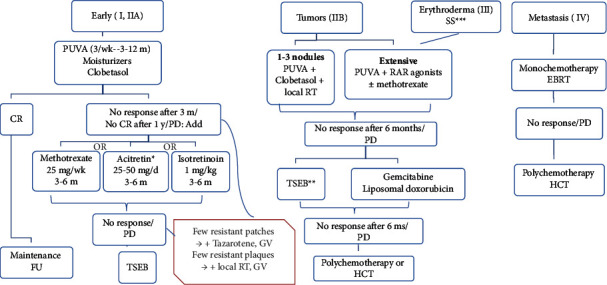
Algorithm for treatment of classic MF/SS. The treatment is presented in a stepwise pattern where patients shift to the next line of therapy in case of the absence of complete response (CR) after 1 year of the current line of therapy, no response after 3–6 months, or the occurrence of progressive disease (PD). For early MF, phototherapy is given where PUVA is more recommended than NB-UVB for our patients with dark skin phenotype (level 2). Potent steroids (level 3) and moisturizers (level 5) are additional basic treatment. Addition of either RAR agonist (level 2) or methotrexate (level 5) is recommended as a second line. Moreover, methotrexate can be combined with acitretin (level 4). TSEB is considered a third line option (level 2). Topical tazarotene (level 3) or gentian violet (level 5) can be added to resistant patches; and localized radiotherapy (level 4) or gentian violet can be added to resistant plaques. Patients with stages (IB-IIA) who show CR should enter a maintenance and follow-up regimen (level 2). For stage IIB with limited disease (up to 3 nodules), localized radiotherapy can be added to SDT (level 4). For stage IIB with multiple nodules, stage III and SS, PUVA plus RAR agonists (level 2) or methotrexate (level 5) or combined methotrexate and acitretin is recommended as a first line (level 4), TSEB is considered a second line option (level 2). Monochemotherapy with gemcitabine or liposomal doxorubicin can be given instead (level 4). Polychemotherapy and allogenic HCT are regarded as a final option (level 3). For stage IV, monochemotherapy is the first line (level 4), followed by polychemotherapy and allogenic stem cell transplantation (level 3). ^*∗*^Acitretin can be combined with methotrexate. ^*∗∗*^TSEB is preferable than monochemotherapy if feasible to the patient. ^*∗∗∗*^Phototesting and slow dose escalation are mandatory in case of erythroderma in stage III and IV. CR: complete response, PD: progressive disease, FU: follow-up, TSEB: total skin electron beam, GV: gentian violet, RT: radiotherapy, RAR: retinoic acid receptor, EBRT: external beam radiotherapy, HCT: hematopoietic cell transplantation.

The main aim of treatment is to improve the patients' quality of life (QOL). It is important to mention that the early use of systemic treatment does not lead to a better outcome than using skin-directed therapy (SDT) [83]. Therefore, treatment options are presented in a stepwise pattern as the main objective of the treatment is to control the patients' disease with minimal toxicity. Therefore, SDT is given as a frontline in patients with early classic MF, while systemic and combined therapy is reserved for the late cases and transformed MF (Figure 3). Monochemotherapy can also be considered for early cases refractory to SDT and other systemic therapies, to decrease the tumour burden and improve the QOL.

Putting patients' feedback into consideration, NB-UVB can be given instead of PUVA if the latter is inaccessible. Moreover, some patients complained of the unavailability of nearby phototherapy units as in rural areas, we suggest shifting to second line in the treatment algorithm as an alternative.

### 3.3. Hypopigmented MF (HMF)

**Figure 4:**
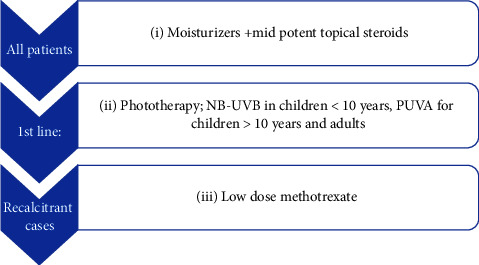
Algorithm for the treatment for hypopigmented MF in Egypt.

Hypopigmented MF (HMF) is the most common variant of MF in children [84–86]. It is common in the Arab population and dark-skinned individuals. Patients usually have other types of MF lesions [87]. Reported treatment includes NB-UVB, PUVA, topical steroids, topical bexarotene, topical tazarotene, and topical carmustine. Phototherapy is the most commonly used treatment and is usually combined with topical steroids [88–90]. PUVA offers greater response and longer remission than NB-UVB [91–93]. Recurrence is common and maintenance PUVA showed lower rate of relapse compared to patients not receiving maintenance treatment [88]. Topical bexarotene and tazarotene are the most common used topical retinoids. Systemic treatment as systemic retinoids and methotrexate has been investigated in adults but there are no sufficient data regarding their use in children [86]. No progression to advanced stage has been noticed [85, 86, 89, 94]; however, Amorim et al. [95] reported progression in some cases. The British Phototherapy Group does not recommend PUVA for children less than 10 years old [96]. Accordingly, phototherapy is recommended as a first line treatment in our institute for HMF (Level 4), where NB-UVB is given to children less than 10 years and PUVA is the preferred treatment modality for patients older than 10 years (level 3). Moisturizers and mid potent steroid are basic treatment for all patients (level 5). Low-dose methotrexate is only reserved for recalcitrant cases (level 5) (Figure 4).

### 3.4. Follicular MF (FMF)

In general, SDT is insufficient to control FMF and multiple systemic therapies are needed [6]. However, it is crucial to differentiate between the indolent early variant and the aggressive advanced variant of FMF [97]. In a single-institution retrospective study, acitretin is used in combination with either radiotherapy or interferon in the treatment of FMF and then continued alone for maintenance [98]. In a case report, 0.1% tazarotene gel and a layer of 0.1% triamcinolone ointment under occlusion were used for resistant lesion of FMF and showed good response [25].

**Figure 5:**
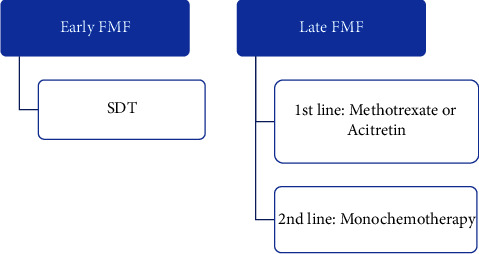
Algorithm for the treatment for FMF. FMF: follicular mycosis fungoides; SDT: skin-directed therapy.

According to NCCN 2020 guidelines, patient with early FMF can be treated with SDT, while those with advanced stage can be treated with category A systemic treatment as methotrexate or acitretin before considering category B systemic treatment as monochemotherapy [13]. This is in agreement with the report of the Dutch cutaneous lymphoma group and other studies that recommend SDT for early FMF cases, while late cases are treated with more intensive treatment [99–101]. Usually, PUVA for early FMF needs longer induction phase [101]. We adhere to the previous regimen in managing FMF (Level 3) (Figure 5).

### 3.5. MF with Large Cell Transformation (LCT)

Large cell transformation (LCT) is diagnosed when large cells constitute more than 25% of lymphoid infiltrates in a skin lesion biopsy. CD30 expression is found in 30% to 50% of transformed cases and these patients may benefit from CD30-directed therapies [8, 102, 103]. However, brentuximab vedotin (anti-CD30) is rarely used in Egypt because it is very expensive and not covered by medical insurance.

MF with LCT is often aggressive and requires systemic treatment (liposomal doxorubicin, pralatrexate, gemcitabine, and romidepsin) [13, 104]. In a single case report, CD30-negative transformed MF showed good response to treatment with bexarotene and methotrexate [105]. Unfortunately, most of the abovementioned treatments are unavailable.

**Figure 6:**
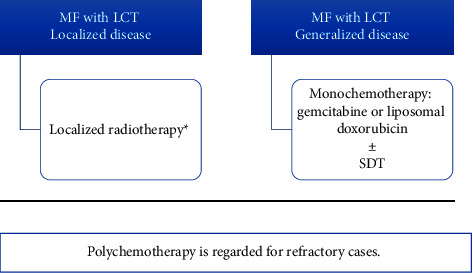
Algorithm for the treatment for MF with LCT. LCT: large cell transformation; SDT: skin-directed therapy. ^*∗*^Other treatment used before transformation can be continued along with radiotherapy.

Accordingly, monochemotherapy (Level 4) as gemcitabine [106] or liposomal doxorubicin [107] can be given in our institute to generalized disease with or without skin-directed therapies [13, 102]. For localized LCT (i.e., restricted to one or few nodules/plaques), localized radiotherapy can be used alone along with the other treatment modalities used before transformation. Polychemotherapy is regarded for refractory cases (Figure 6) [13].

### 3.6. Maintenance Therapy and Follow-Up Schedule

Though previous reports did not show solid evidence for the use of maintenance therapy with phototherapy [108], a multicentre prospective randomized clinical trial with low-dose, low-frequency PUVA maintenance regimens showed prolonged median disease-free remission. Patients with CR were randomized to receive PUVA maintenance for 9 months. This prolongs the median disease-free remission from 4 months to 15 months (Level 2) [109]. Maintenance NB-UVB can also prolong the remission in the early stage [110]. Moreover, in a recent review, maintenance therapy was found to be necessary in most of the cases [111].

**Figure 7:**
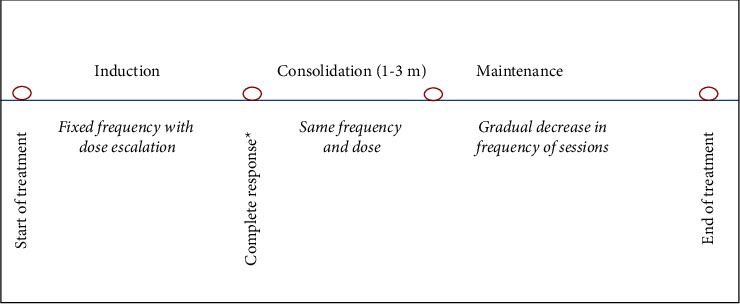
Maintenance regimen with phototherapy. Treatment of MF patients with phototherapy includes 3 phases: induction, consolidation, and maintenance. The dose of UV is escalated during the induction phase with fixation of the frequency of sessions, while both the dose and the frequency are fixed during the consolidation phase. During the maintenance phase, there is a gradual decrease of the frequency of the sessions with fixation of the dose [38]. ^*∗*^Complete response is determined clinically, and has to be  ≥ 4 weeks; biopsy is only required when assessing postinflammatory hyperpigmentation or erythema versus the presence of residual lesion [112].

According to the guidelines for phototherapy of MF/SS of the United States Cutaneous Lymphoma Consortium (US CLC) [38], maintenance therapy by either NB-UVB or PUVA is given to induce prolonged remission off therapy in MF patients that have potential risk of decreased survival. They defined three phases of phototherapy: induction, consolidation, and maintenance phases. Induction phase represents the time from the start of phototherapy till CR is achieved. Consolidation phase lasts for 1–3 months after CR. Maintenance phase follows the consolidation phases till discontinuation of therapy (Figure 7). Accordingly, we follow these US CLC guidelines. Recommendations for the UV doses and the frequency of sessions during the induction and maintenance phases are included in (Supplementary Table 6–8 available topical treatment).

For advanced MF/SS, salvage PUVA therapy can be used after systemic therapy [31]. Other reported maintenance therapies include low-dose methotrexate, acitretin, tazarotene, and topical steroids (level 5) [10].

Follow-up is recommended every month during induction, consolidation, and maintenance stages, then every 3 months afterwards.

## 4. Conclusion

We suggest a practical algorithm for the treatment of MF in patients with darker phenotype, in adherence with the international guidelines and in the context of limited medical resources.

## Figures and Tables

**Table 1:** Available topical treatment for MF/SS in Egypt.

Topical treatment	Type of the study	Stage	Response rate, duration of remission	OCEBM
Corticosteroid [20]	Prospective (uncontrolled)	IA-IB	IA–IB 94%	Level 3
Tazarotene gel [23] (refractory lesions)	Prospective open-label	Early patch <20% BSA	58% achieved at least a moderate (>50%) global improvement in BSA	Level 3
Tazarotene cream (monotherapy) [25]	Prospective open-label	IA to IIA	60% CR, for 6 months in 83%	Level 3
Moisturizer [31]	RCT for peldesine vs placebo (mechanism-based)	IA to IIA	28% vs 24% placebo	Level 5
Gentian violet	In vitro [27] case report [28]	CTCL lines IB	4–6 apoptosis in CTCL lines > normal KC PR	Level 5

OCEBM: Oxford Centre for Evidence-Based Medicine, BSA: body surface area, CR: complete response, RCT: randomized controlled trial, CTCL: cutaneous T cell lymphoma, KC: keratinocyte, PR: partial response.

**Table 2:** Phototherapy studies for MF.

Phototherapy	Type of the study	Stage	Response rate, duration of remission	OCEBM
NB-UVB [36]	Prospective	I-IIA	CR in 100% patch vs 60% plaques	Level 3
NB-UVB [41]	Prospective	I	CR in 75%, time to relapse 4.5 m	
NB-UVB [40]	Retrospective	I	84%	
NB-UVB [51]	Retrospective	I	84% (IA), 78% (IB)	
PUVA [46]	Retrospective	I-IIA	CR 63%, 33% maintained remission (84 m), 33% relapsed with DFI (39 m)	Level 2
PUVA [52]	Retrospective	IA-IB	95%, 43 m	
PUVA [53]	Prospective	IA-IB	100%, 20 m (IA), 17 m (IB)	
PUVA versus NB-UVB [54]	Retrospective	PUVA: IA-IVANB-UVB: IA-IIB	Remission 85% vs 83% time to relapse 10 m vs 11.5 m	Level 2
PUVA versus NB-UVB [55]	Retrospective	I-IIA	Remission: 87.4% vs 94.7% time to relapse: 11.5 m vs. 14.0 m No significant difference	
PUVA versus NB-UVB [44]	Meta-analysis	I-IIA	CR in 73.8% vs 62.2% significant difference	

OCEBM: Oxford Centre for Evidence-Based Medicine, CR: complete response, DFI: disease-free interval.

**Table 3:** Protocol of monochemotherapy treatment for MF/SS in Egypt.

Monochemotherapy	Regimen	Stage	OCEBM
Methotrexate [8, 13, 79, 80]	25–50 mg/week for 3–6 m	IIB, III, SS 2^nd^: IA-IIA	Level 4
Gemcitabine [8, 13, 79, 81]	1200 mg/m2 IV on days 1, 8, 15 of a 28-day schedule for 3–6 m	IV 2^nd^: IIB, III	Level 4
Pegylated liposomal doxorubicin [8, 13, 80, 82]	20–30 mg/m2 IV, every 3-4 weeks for 12–24 weeks	IV 2^nd^: IIB, III	Level 3

OCEBM: Oxford Centre for Evidence-Based Medicine.

## Data Availability

The data supporting these guidelines are from previously reported studies, which have been cited. The processed data include questionnaires from the patients that are available from the corresponding author upon request.

## Supplementary Materials

Supplementary Materials Supplementary Table 1: ISCL/EORTC revision of the TNMB classification of MF/SS (2007). Supplementary Table 2: histopathologic staging of lymph nodes in MF/SS (2007). Supplementary Table 3: ISCL/EORTC revision to the staging of MF/SS (2007). Supplementary Table 4: search terms used in the literature review of the current study. Supplementary Table 5: details of OCEBM and identification of the level of evidence using OCEBM (2011). Supplementary Table 6: treatment recommendations for NB-UVB induction phase in patients with MF based on Fitzpatrick skin type. Supplementary Table 7: treatment recommendations for PUVA induction phase in patients with MF based on Fitzpatrick skin type. Supplementary Table 8: maintenance phase treatment of patients with MF with UV light (based on a thrice-weekly induction schedule for NB-UVB phototherapy and at least twice weekly for PUVA phototherapy).
