# Supplementary material for: Suggested Guidelines for the Treatment of Mycosis Fungoides in Countries with Limited Resources
Source: Dermatol Res Pract. 2023 Jan 31;2023:1360740. doi: 10.1155/2023/1360740 (PMC9904957; doi:10.1155/2023/1360740)
Supplement: Supplementary Materials — Supplementary Table 1: ISCL/EORTC revision of the TNMB classification of MF/SS (2007). Supplementary Table 2: histopathologic staging of lymph nodes in MF/SS (2007). Supplementary Table 3: ISCL/EORTC revision to the staging of MF/SS (2007). Supplementary Table 4: search terms used in the literature review of the current study. Supplementary Table 5: details of OCEBM and identification of the level of evidence using OCEBM (2011). Supplementary Table 6: treatment recommendations for NB-UVB induction phase in patients with MF based on Fitzpatrick skin type. Supplementary Table 7: treatment recommendations for PUVA induction phase in patients with MF based on Fitzpatrick skin type. Supplementary Table 8: maintenance phase treatment of patients with MF with UV light (based on a thrice-weekly induction schedule for NB-UVB phototherapy and at least twice weekly for PUVA phototherapy). [file 1360740.f1.docx]

**Supp Table 1** ISCL/EORTC revision of the classification of MF/SS (2007)

| TNMB |  |
| --- | --- |
| **Skin** |  |
| T_1_ | Limited patches, papules, and/or plaques covering < 10% of the skin surface. May further stratify into T_1a_ (patch only) vs T_1b_ (plaque ± patch). |
| T_2_ | Patches, papules or plaques covering ≥ 10% of the skin surface. May further stratify into T_2a_ (patch only) vs T_2b_ (plaque ± patch). |
| T _3_ | One or more tumors (≥ 1-cm diameter) |
| T _4_ | Confluence of erythema covering ≥ 80% body surface area |
| **Node** | ***See Supp Table 2*** |
| N _0_ | No clinically abnormal peripheral lymph nodes; biopsy not required |
| N _1_ | Clinically abnormal peripheral lymph nodes; histopathology Dutch grade 1 or NCI LN_0-2_ |
| N _2_ | Clinically abnormal peripheral lymph nodes; histopathology Dutch grade 2 or NCI LN_3_ |
| N _3_ | Clinically abnormal peripheral lymph nodes; histopathology Dutch grades 3-4 or NCI LN_4_; clone positive or negative |
| **Visceral** |  |
| M _0_ | No visceral organ involvement |
| M _1_ | Visceral involvement (must have pathology confirmation and organ involved should be specified) |
| **Blood** |  |
| B 0 | Absence of significant blood involvement: ≤ 5% of peripheral blood lymphocytes are atypical (Sézary) cells |
| B 1 | Low blood tumor burden: > 5% of peripheral blood lymphocytes are atypical (Sézary) cells but does not meet the criteria of B_2_ |
| B 2 | High blood tumor burden: ≥ 1000/μL Sézary cells with positive clone |

ISCL/EORTC: International Society for Cutaneous Lymphomas/ European Organization for Research and Treatment of Cancer, MF: mycosis fungoides, SS: Sézary syndrome, NCI LN: National Cancer Institute - Lymph Nodes

**Supp Table 2** Histopathologic staging of lymph nodes in MF/SS (2007)

| Updated ISCL/EORTC classification | Dutch system | NCI-VA classification |
| --- | --- | --- |
| N_1_ | Grade 1: dermatopathic lymphadenopathy (DL) | LN_0_: no atypical lymphocytes |
|  |  | LN_1_: occasional and isolated atypical lymphocytes (not arranged in clusters) |
|  |  | LN_2_: many atypical lymphocytes or in 3-6 cell clusters |
| N_2_ | Grade 2: DL; early involvement by MF (presence of cerebriform nuclei > 7.5 μm) | LN_3_: aggregates of atypical lymphocytes; nodal architecture preserved |
| N_3_ | Grade 3: partial effacement of LN architecture; many atypical cerebriform mononuclear cells (CMCs); Grade 4: complete effacement | LN_4_: partial/complete effacement of nodal architecture by atypical lymphocytes or frankly neoplastic cells |

ISCL/EORTC: International Society for Cutaneous Lymphomas/ European Organization for Research and Treatment of Cancer, MF: mycosis fungoides, SS: Sézary syndrome, NCI VA: National Cancer Institute–Veterans Administration, LN: lymph node

**Supp Table 3** ISCL/EORTC revision to the staging of MF/SS (2007)

|  | T | N | M | B |
| --- | --- | --- | --- | --- |
| IA | 1 | 0 | 0 | 0,1 |
| IB | 2 | 0 | 0 | 0,1 |
| IIA | 1,2 | 1,2 | 0 | 0,1 |
| IIB | 3 | 0-2 | 0 | 0,1 |
| III | 4 | 0-2 | 0 | 0,1 |
| IIIA | 4 | 0-2 | 0 | 0 |
| IIIB | 4 | 0-2 | 0 | 1 |
| IVA_1_ | 1-4 | 0-2 | 0 | 2 |
| IVA_2_ | 1-4 | 3 | 0 | 0-2 |
| IVB | 1-4 | 0-3 | 1 | 0-2 |

**Supp Table 4** Search Terms

| “mycosis fungoides”, “hypopigmented mycosis fungoides”, “transformed mycosis fungoides”, “folliculotropic mycosis fungoides”, “corticosteroid(s)”, “phototherapy”, “PUVA”, “psoralen-UVA”, “UVB”, “ultraviolet B”, “moisturizers”, “retinoid(s)”, “acitretin”, “isotretinoin”, “tazarotene”, “radiotherapy”, “total skin electron beam therapy”, “TSEB”, “chemotherapy”, "methotrexate”, “gemcitabine”, “liposomal doxorubicin”, “CHOP”, “EPOCH”, “CVP”, “CAVE”, “systemic treatment”, “combined treatment”, “maintenance”, “antibiotic”, “decolonization”, “mupirocin”, “gabapentin”, “mitrazapine”, “aprepitant” “prednisone”, “guidelines”, “meta-analysis”, “systemic reviews”, “randomized controlled trials”, “RCT”, “non-randomized controlled trial”, “case control”, “cohort”, “case series”, “case report”. |
| --- |

**Supp Table 5** Oxford Centre for Evidence-Based Medicine 2011 Levels of Evidence (OCEBM)

| **Question: Does this intervention help?** | **Level ^a^** |
| --- | --- |
| Systematic review of randomized trials or *n*-of-1 trials | 1 |
| Randomized trial or observational study with dramatic effect | 2 |
| Non-randomized controlled cohort/follow-up study ^b^ | 3 |
| Case series, case-control studies, or historically controlled studies ^b^ | 4 |
| Mechanism-based reasoning | 5 |

^a^ Level may be graded down on the basis of study quality, imprecision, indirectness (study PICO does not match questions PICO), because of inconsistency between studies, or because the absolute effect size is very small; Level may be graded up if there is a large or very large effect size.

^b^ As always, a systematic review is generally better than an individual study.

**Supp Table 6** Treatment recommendations for narrowband ultraviolet B light induction phase in patients with mycosis fungoides based on Fitzpatrick skin type

| **Skin type** | **Initial dose (mJ/cm^2^)** | **Increments (by mJ/cm^2^)** |
| --- | --- | --- |
| I | 130 | 15 |
| II | 220 | 25 |
| III | 260 | 40 |
| IV | 330 | 45 |
| V | 350 | 60 |
| VI | 400 | 65 |

If no response after 20 treatments, may increase exposure by additional 50 to 100 mJ/cm**^2^** above the previous incremental increase.

In early-stage disease, it is generally acceptable to cover the face during phototherapy when the face is not involved.

**Supp Table 7** Treatment recommendations for psoralen plus ultraviolet A light phototherapy induction phase in patients with mycosis fungoides based on Fitzpatrick skin type

| **Skin type** | **Initial dose (J/cm^2^)** | **Increments (by J/cm^2^)** |
| --- | --- | --- |
| I | 0.5 | 0.5 |
| II | 1.0 | 0.5 |
| III | 1.5 | 1.0 |
| IV | 2.0 | 1.0 |
| V | 2.5 | 1.5 |
| VI | 3.0 | 1.5 |

If no response after 20 treatments, increase exposure by additional 0.5 to 1 J cm**^2^** above the previous incremental increase. Patients with erythroderma must be treated cautiously and treatments should be spread out or lower increments of increase over last treatment used if any pain, stinging, or increase in erythema occurs.

In early-stage disease, it is generally acceptable to cover the face during phototherapy when the face is not involved.

**Supp Table 8** Maintenance phase treatment of patients with mycosis fungoides with ultraviolet light (based on a thrice-weekly induction schedule for narrowband ultraviolet B light phototherapy and at least twice weekly for psoralen plus ultraviolet A light phototherapy)

|  | **NB-UVB** | | **PUVA** | |
| --- | --- | --- | --- | --- |
| **Treatment frequency** | **Weeks** | **Dose relative to end consolidation** | **Weeks** | **Dose relative to end consolidation** |
| **Twice weekly** | 4-8 | Same | 4-8 | Same |
| **Weekly** | 4-8 | Same | 4-8 | Same |
| **Every 10 days** | 4-8 | Same | 4-8 | Same |
| **2 wks*** | 4-8 | Decrease by 25% | 4-8 | Same |
| **3 wks** | NA | Decrease by 50% | 4-8 | Same |
| **4 wks** | NA | NA | 4-8 | Same |

NA, Not applicable.

*Recommended treatment after complete response is 3 months, including a consolidation period

with complete response being documented before transitioning to less frequent treatments.
